# Magnitude and Determinants of Needlestick and Sharp Injuries among Nurses Working in Tikur Anbessa Specialized Hospital, Addis Ababa, Ethiopia

**DOI:** 10.1155/2020/6295841

**Published:** 2020-12-17

**Authors:** Bikis Liyew, Menbeu Sultan, Mebrat Michael, Ambaye Dejen Tilahun, Tilahun Kassew

**Affiliations:** ^1^School of Nursing, College of Medicine and Health Sciences, Department of Emergency and Critical Care Nursing, University of Gondar, Gondar, Ethiopia; ^2^Department of Emergency Medicine and Critical Care, St. Paul's Hospital Millennium Medical College, Addis Ababa, Ethiopia; ^3^School of Medicine, College of Medicine and Health Science, Addis Ababa University, Addis Ababa, Ethiopia; ^4^Department of Psychiatry, College of Medicine & Health Sciences, University of Gondar, Gondar, Ethiopia

## Abstract

**Background:**

Needlestick and sharp injuries are a big risk to the health of nurses. Every day, nurses face the likelihood that they will injure themselves. Although many injuries will have no adverse effect, the possibility of acquiring infections like hepatitis C virus, hepatitis B virus, and human immunodeficiency virus can cause untold psychological harm. Nurses are in danger of injuries caused by needlestick and sharp instruments in hospitals.

**Objective:**

The objective of this study was to assess the magnitude and determinants of needlestick and/or sharp injuries among nurses working at Tikur Anbessa Specialized Hospital, Addis Ababa, Ethiopia, 2018.

**Methods:**

An institution-based cross-sectional study was conducted among 268 nurses working at Tikur Anbessa Specialized Hospital from February to March 2018. A stratified random sampling technique was used to select the study participants. Data were collected using a self-administered questionnaire. A bivariate and multivariate logistic regression model was fitted to spot factors associated with needlestick and/or sharp injury. An adjusted odds ratio with a 95% confidence interval was computed to determine the level of significance.

**Result:**

The prevalence of needlestick and/or sharp injuries among nurses was 36.2% (95% CI 30.2%, 42.3%). Presence of contaminated needles and/or sharp materials in the working area (AOR = 2.052 (95% CI 1.110, 3.791)), needle recapping after use (AOR = 1.780 (95% CI 1.025, 3.091)), working in the pediatric ward (AOR = 0.323 (95% CI 0.112, 0.930)), and being female (AOR = 0.461 (95% CI 0.252, 0.845)) were significantly associated with needlestick and/or sharp injury at *p* value of ≤0.05. *Conclusion and Recommendation*. The proportion of needlestick and/or sharp injury was high among nurses. The safety of nurses depends directly on the degree to which nurses can identify and control the numerous occupational hazards specific to jobs. Thus, working unit specific safety precautions, a safe working environment, and appropriate needle and sharp disposal improve nurses' safety practices and thereby decrease the injuries.

## 1. Introduction

Needlestick and sharp injuries are wounds that are caused by sharps that accidentally puncture the skin. Sharps include hypodermic needles, blood collection needles, and IV (intravenous) cannulas or needles as well as items such as scalpels, razor blades, lancets, retractors, scissors, pins, clamps, cutters, staples, and glass items [1]. These preventable injuries expose health care workers to over 20 different bloodborne pathogens, which resulted in 1000 infections per year [2]. It is estimated that through occupational exposure, 2.6% of health care workers are exposed to hepatitis C virus (HCV), 5.9% to hepatitis B virus (HBV), and 0.5% to HIV annually. This equates to approximately 16,000 HCV infections, 66,000 HBV infections, and 200-600 HIV (human immunodeficiency virus) infections worldwide [3]. According to the World Health Organization (WHO), needlestick and sharp injuries cause about 40% of hepatitis C and B infections and 2.5% of HIV infections among health care providers worldwide [4]. The World Health Organization has also estimated that in developing regions, 40%–65% of HBV and HCV infections in HCWs are attributable to percutaneous occupational exposure [5]. Health care workers (HCWs) in Africa suffer two to four needlestick injuries per year on average [6], with Nigeria, Tanzania, and South Africa reporting 2.1% injuries per year on average [7]. The Centers for Disease Control and Prevention (CDC) estimates that about 236,000 to 384,000 hospital workers sustain needlestick and sharp injuries, and nurses share 40% of it [8]. In the United States of America (USA), it was estimated that the reported incidence of NSI among nurses is currently 16.3% [9]. 48% of nurses in Ireland [10], 39% of registered nurses in the USA [11], 39.4% of nurses in Iran [12] reported an incident in their careers by a needle or sharp injury in the last 12 months.

Globally, it is estimated that 3 million health care workers worldwide experience NSI every year; of those, up to 50% of all NSI are being sustained by nurses [13]. Previous works of literature conducted in South Korea (70.4%) [14], Pakistan (67%) [15], Thailand (55.5%) [16], India (33.3%) [17], Nepal (74%) [18], Iran (41%, 54%) [19, The World Health Organization (WHO) reports that the number of needlestick injuries per person among health care staff is 4 per year in Africa, Western Mediterranean, and Asia [23]. Developing countries, especially those in sub-Saharan Africa, account for the highest prevalence of HIV-infected patients, and more than 90% of occupational exposure occurs in these countries [24–27]. The burden of the problem is not only on individual health but also on human resources and economic and social destruction [28]. Bloodborne pathogens are generally considered endemic in sub-Saharan Africa [29]. For instance, 62% of nurses in Nigeria [30], 18.8% in South Africa [31], 74.57% in Egypt [1], and 40.2% in Nigeria had sustained a needlestick injury in 12 months [30].

Moreover, health care workers practicing in developing countries such as Ethiopia are more exposed to human immunodeficiency virus (HIV) and hepatitis B virus (HBV) following occupational exposure and are less likely to use postexposure prophylaxis (PEP) than those working in developed countries [32, 33]. Even though there is no national data regarding the magnitude of NSSI among HCWs, particularly among nurses, the prevalence of sharp injuries among HCPs was 32.8% [34]. In studies conducted in Southwest Ethiopia (58.8%) [35]; Jimma University Hospital, Ethiopia (39.3%) [36]; and Bahir Dar, Ethiopia (66.6%) [37], nurses sustained sharp and or needlestick injuries in the past 12 months. Needlestick and sharp injuries represent a serious hazard within the health care industry, with professional nurses incurring an outsized proportion of the entire burden particularly with items that have been previously used on patients [38–40].

These injuries occur in different types of procedures such as needle recapping, operative procedures, blood collection, intravenous line administration, suturing, checking blood sugar, and poor sharp disposal [41]. The causes include various factors: type and elegance of needles, recapping activity, handling/transferring specimens, collision between HCWs and sharps, during cleanups, manipulating needles in patient line-related work, passing/handling devices, or failure to dispose of the needle in puncture-proof containers [42]. Therefore, needlestick and sharp injuries are a serious concern to all health care personnel and pose a significant risk of transmission of occupational bloodborne pathogens [43]. There is a paucity of information in Ethiopia particularly in Tikur Anbessa Specialized Hospital describing the magnitude and determinants of needlestick and sharp injuries. In Ethiopia, where primary health care services are covered by nurses, the risk of contracting infections following needlestick and sharp injuries is high in their day-to-day activities. Since the researchers have seen different kinds of literature, there is no single study on nurses other than overall health professionals in the study area, and the devastating effect of NSSI in the economic and psychological terms and health of nurses and their families needs more attention in the daily life of health care professionals. The reason why Tikur Anbessa Specialized Hospital was selected was that this hospital is the largest referral hospital in the country. It is also an establishment where specialized clinical services that are not available in other public or private institutions are rendered to the entire nation. Hence, the objective of this study is to determine the magnitude and determinants of needlestick and/or sharp injuries among nurses at Tikur Anbessa Specialized Hospital.

### 1.1. Conceptual Framework

**Figure 1:**
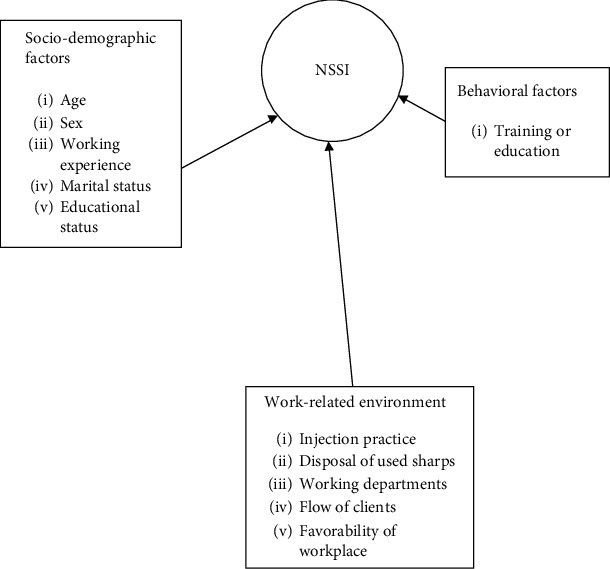
Conceptual framework literature review, TASH, Addis Ababa, Ethiopia, June 2018.

The factors associated with needlestick and sharp injuries are classified as sociodemographic factors like sex, age, service year or experiences, and marital status; work-related environmental factors like injection practice, disposal of used sharps, department, and favorability of workplace; and behavioral factors like education and training. The three factors are interrelated to affect one another.  Figure 1 shows the interrelation between the sharp and needlestick injury (independent) variables in detail [32, 33, 44].

## 2. Methods

### 2.1. Study Setting

The study was conducted in Tikur Anbessa Specialized Hospital which is found in Addis Ababa (the capital city of Ethiopia) in the Lideta subcity. According to the Central Statistical Agency (CSA) of Ethiopia, as of 2013, the town of Addis Ababa has a total population of 3,130,673, of which 1,478,890 are men and 1,624,783 are women. It is the nation's largest and highest referral hospital. This hospital sees approximately 370,000–400,000 patients a year, but the exact number is not known. It has 700 beds. This is the largest teaching hospital in Ethiopia. There are a total of 789 nurses with different qualifications. The hospital is planned and accommodated and facilitated with the outpatient department (OPD), which has seven X-ray, nine surgical, and two diagnostic laboratory rooms. The hospital provides medical services in internal medicine, gynecologic and obstetric, surgical, pediatric, and emergency departments. The hospital also has special units (referral clinics), which include chest, renal, neurology, cardiology, dermatology, orthopedic, general surgical, gynecologic and obstetric, diabetic, hematology, and medical intensive care units and units for sexually transmitted diseases, gastrointestinal diseases, and infectious diseases.

### 2.2. Study Design and Populations

An institution-based cross-sectional study was conducted to assess the prevalence and associated factors of needlestick and/or sharp injuries (NSSI) from February 19 to March 31, 2018. The source population for this study was all nurses who were working in Tikur Anbessa Specialized Hospital. The sample populations of the study were all selected nurses working in Tikur Anbessa Specialized Hospital at the time of data collection. Nurses either males or females who were working in the same department or unit for at least one year and all those registered nurses who were working in Tikur Anbessa Specialized Hospital of Addis Ababa during the study period involved in clinical work were included in the study, whereas the nursing personnel not involved in the direct management of the patients (e.g., nursing managers, tutorial staff) and those nurses who were students, retired, and on sick or maternity leave and with less than six months of working experience were excluded from the study.

### 2.3. Sample Size and Sampling Procedure

**Figure 2:**
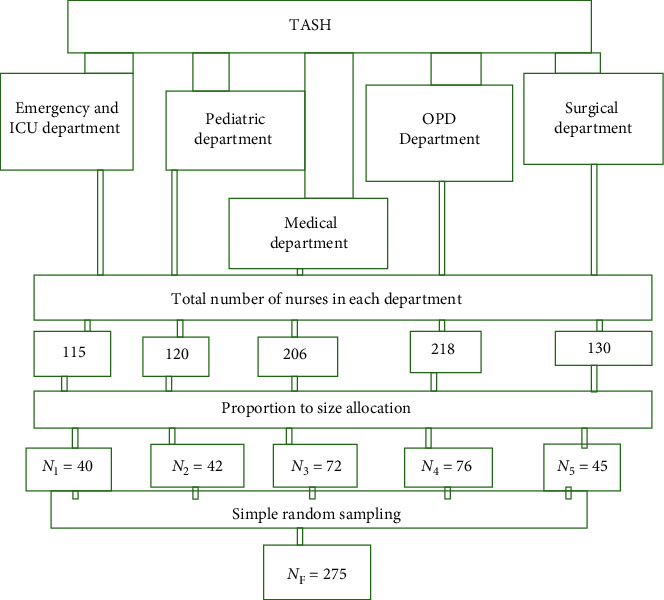
Schematic presentation of the sampling procedure to select the study participants, TASH, Addis Ababa, Ethiopia, June 2018.

The actual sample size for the study was determined using the formula for a single population proportion. To determine the initial sample size, the following assumption has been made: *n*_i_ is the initial sample size from a finite population, *n*_f_is the final sample size from a finite population, *Z* is the standard score (critical value) corresponding to the 95% confidence level, and *p* is the proportion of nurses experiencing needlestick and sharp injuries, which was 39.3%, taken from a study done in Jimma University Specialized Hospital taking the prevalence of NSSI among nurses [45]. So sample size can be calculated as follows: *n*_i_ = (*Z*_a_/2)2 × *P*(1 − *P*)/*d*2 = (1.96)2 × 0.393(1 − 0.393)/(0.05)2 = 367. Because of the total population size of the study area (*N* = 789) which is less than 10,000, we shall apply the population correction formula: *n*_f_ = *n*_i_/1 + *n*_i_/*N* = 367/1 + 367/789 = 250, and by adding a 10% nonresponse rate, the total sample size of this study was 275. A stratified random sampling technique was used to select the nurses. Hospital departments are classified into 5 main strata that had nearly the same working conditions: (1) internal medicine, (2) pediatric, (3) surgical, (4) outpatient clinic, and (5) emergency and intensive care departments. The proportional allocation was taken from each stratum. Data were collected in Tikur Anbessa Specialized Hospital by introducing themselves (researchers), explaining the aim of the study, and acquiring consent. Data assurance was applied from the very beginning by review prior to the study and pretested by taking 5% of the study sample and by closely monitoring the activity of data collectors and supervisors by the principal investigator. The collected data were checked for completeness, accuracy, and clarity. Codes were given to the questionnaire, and any identified errors could get traced back using the codes. Each filled questionnaire was checked and reviewed for completeness by the supervisor and principal investigator; the necessary feedback was given to the data collectors the next morning (Figure 2).

### 2.4. Data Collection Tool and Procedure

Data were collected using a structured self-administered questionnaire. The questionnaire was adapted by reviewing the literature of similar studies on needlestick and sharp injuries [46–48] and adapted from the WHO/ICN (World Health Organization and International Council of Nurses) tool kit injection safety and the experience of the research done by the Ethiopian Nurse Association [49]. The questionnaire had 3 parts:

*Part one*: sociodemographic characteristics and work-related aspects (years of experience, department, hepatitis B vaccine status, etc.)

*Part two*: information on needlestick injury and sharp injury

*Part three*: circumstances or procedures that contribute to NSSI

For a binary outcome variable indicating “have you had any sharp and needlestick injury since last year?”, the response was coded as “yes” and “no” and it was used as the dependent variable. Data were collected by four trained data collectors (BSC nurse professionals) using the Amharic version of the questionnaire. The questionnaire was designed in English and was translated to Amharic, the official language of Ethiopia, and back to English (forward and backward translation for its consistency). The training was on introduction to NSSI, research methods, sampling and recruitment, and ethical aspects of research.

### 2.5. Data Processing and Analysis

The data cleanup and cross-checking were done before analysis. Data were checked and coded, and completed questionnaires were given identification numbers and entered into Epi Info version 7.2.2; then, they were exported to SPSS version 23 for analysis. Both the descriptive and analytical statistical procedures were utilized. Descriptive statistics like percentage, mean, and standard deviation were used for the presentation of sociodemographic data and prevalence of needlestick and/or sharp injury. Tables were also used for data presentation. Binary logistic regression was used to identify associated factors of needlestick and sharp injuries among nurses working at Tikur Anbessa Specialized Hospital. All explanatory variables with a *p* value of ≤0.2 from the bivariate logistic regression model were fitted into the multivariate logistic regression model to control the possible effect of confounders, and finally, the variables which had an independent association with needlestick and/or sharp injury were identified based on OR with 95% CI, and *p* values less than 0.05 were significant. The total model was significant (*p* ≤ 0.001). Multicollinearity was detected by tolerance, variance inflation factor (VIF), and correlation matrix. In this study, the values of tolerance, variance inflation factor (VIF), and correlation matrix were 0.498-0.964, 1.037-2.009, and ≤0.66, respectively.

The value of the standard error in the model (0.127) was below 5 which indicated no multicollinearity among variables. The result of the Hosmer and Lemeshow test (*p* = 0.791) indicated the goodness of fit of the model. Nagelkerke's *R*^2^ shows that about 50% of the variation in the outcome variable (NSSI) is explained by this binary logistic regression model.

## 3. Result

### 3.1. Sociodemographic Characteristics of Participants

A total of 268 hospital nurses responded fully to the self-administered questionnaire providing a response rate of 97.5%. Of the total, 191 (71.3%) respondents were female nurses. 115 (42.9%) of respondents were between the ages of 25-30 with a mean age of 29.97 years (SD ± 5.68 years). One hundred eighty-seven (69.8%) of participants were Orthodox Christians. More than half (158 (58.9%)) of participants were single. One hundred ninety-eight (73.9%) of nurses have work experience of fewer than five years with a mean work experience of 5.08 years (SD ± 5.883 years). The majority (202 (75.4%)) of the study participants were BSC nurses. Regarding the injection environment, 187 (69.8%) staff responded that their injection environments were unsafe. From the total respondents, one hundred forty-six (54.5%) had offsite and onsite training on infection prevention before the study (Table 1).

### 3.2. Magnitude and Circumstances of Needlestick and/or Sharp Injuries

The prevalence (occurrence) of needlestick and sharp injuries to nurses in TASH was 97 (36.2%) (95% CI 30.2%, 42.3%). From the total of respondents who had experienced NSSI in the last 12 months before the study, 49.5% were exposed once, while 27.8%, 14.4%, and 8.2% were exposed two, three, and four times per year, respectively. But out of the total respondents who had experienced NSSI in the last year, 58.8% of nurses were exposed one month before the study, while 6.2% were exposed two times and none of the respondents were exposed three, four, and more times per month. Nearly one-half of (46.2%) injuries occurred in the ICU. Other injuries occurred in the surgical (44.4%), medical (39.9%), emergency (36%), OPD (35.6%), and pediatric (20%) departments. Regarding parts of the body injured, the finger accounted for 72.2% followed by the hand (15.5%), and 7.2%, 7.2%, and 6.2% were the arm, thigh, and palm, respectively.

**Figure 3:**
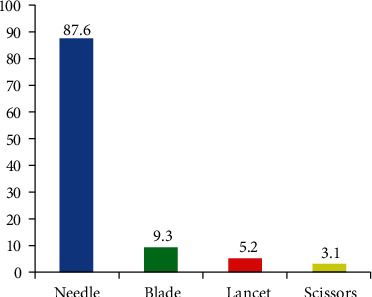
Frequency distribution of causative tools of needlestick and sharp injuries among exposed nurses during the last year working at TASH, Addis Ababa, Ethiopia, June 2018.

The degree or severity of injury accounted for by slight skin penetration was 53.6%, followed by superficial and deep injuries which were 33% and 18.6%, respectively. On the other hand, injuries of 77.3% of nurses were self-inflicted and injuries of 14.4% and 12.4% of nurses were inflicted by another staff and a noncompliant patient, respectively. Regarding the practice of nurses on the job, 44% of the respondents had recapped needles after use at least once during their work time. Of those, 69.5% of the needles were recapped using one-handed recapping, whereas nearly one-third (30.5%) of needles were recapped using two-handed recapping. Among those nurses exposed to NSSI, workload (61.9%), fatigue (7.2%), and lack of proper equipment disposal (35.1%) were perceived as causes of NSSI. 64.5% of nurses know in which department or room they report, and 71.3% of nurses responded that safety boxes were available at the right working places. The study result revealed that the most frequent causative tools of needlestick and sharp injuries among exposed nurses were needles (87.6%) followed by blades (9.3%) and then lancets (5.2%) (Figure 3).

This study result represented that the most frequent procedures at which exposure happens were injection, sample drawing, and operation (38.1%, 24.7%, and 16.5%), respectively. In this study, 51.5% of nurses used antiseptic after NSSI exposure while 45.4% let blood flow. Regarding vaccination, only 2.1% took the vaccine while no one reported the incident after exposure (Table 2).

**Figure 4:**
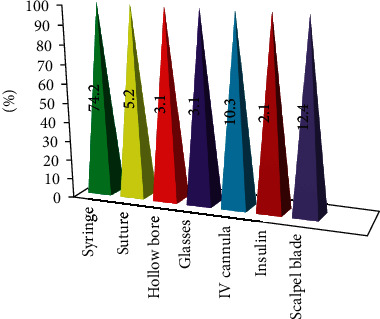
Frequency distribution of the type of items that causes NSSI among exposed nurses during the last year working at TASH, Addis Ababa, Ethiopia, June 2018.

In this study, among exposed nurses during the last year, the most likely cause of needlestick and/or sharp injuries was the syringe (74.2%) (Figure 4).

**Figure 5:**
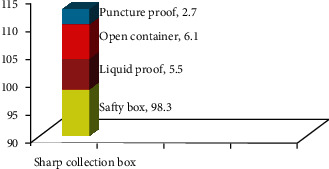
Distribution of the sharp collection box in the clinical area of TASH, Addis Ababa, Ethiopia, June 2018.

In this study result, the factors that contributed to NSSI were excess clients (35.7%) followed by shortage of gloves (29.6%), and the rest were suturing (17.35%), shortage of sharp collection boxes (13.3%), emergencies (5.1%), recapping of used needles (8.2%), and removing of used needles (12.2%). In another way from the total respondents, 193 (72%) of nurses observe needlestick and sharp injuries on nurses. From this, in 101 (52.3%) nurses, NSSI occurs by the abrupt movement of patients during clinical practice, followed by unsafe sharp collection (51 (26.4%)), and the rest were two-handed recapping (37 (19.2%)) and carelessness (negligence) (18 (9.3%)) of nurses, respectively. In this study, from 268 participants, 180 (67.2%) of nurses say that there was a sharp collection box in the clinical area (Figure 5).

**Figure 6:**
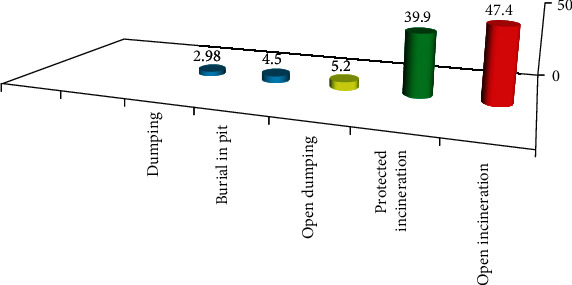
Percentage distribution of the needle, syringe, and sharp disposal system in TASH, Addis Ababa, Ethiopia, June 2018.

In this study, from 268 study participants, 188 (70.1%) of nurses had seen overfilled sharp collection containers in the clinical area, whereas 50 (18.7%) and 30 (11.2%) had seen torn needles and seen dirty syringes inside, respectively (Figure 6).

### 3.3. Determinants of Needlestick and/or Sharp Injuries within the Past 12 Months

#### 3.3.1. Bivariate Logistic Regression Model Analysis of Factors Associated with NSSI

As shown in Table 3, the regression analysis of sociodemographic and NSSI circumstances on bivariate logistic regression showed that sex (COR = 0.425 (95% CI 0.247, 0.731)), educational status (COR = 0.227 (95% CI 0.075, 0.688)), current working department (COR = 0.313 (95% CI 0.118, 0.8270)), dirty sharps in working places (COR = 1.939 (95% CI 1.098, 3.423)), and needle recapping (COR = 1.964 (95% CI 1.185, 3.255)) had a significant association with NSSI. On bivariate logistic regression analysis, respondents being diploma holders in educational status were 77% less likely to risk experiencing injury compared to their counterparts (Table 3).

#### 3.3.2. Multivariate Logistic Regression Model Analysis of Factors Associated with NSSI

In the multivariate logistic regression analysis, sex, current working department, dirty sharps in working places, and needle recapping are statistically significant with the occurrence of needlestick and/or sharp injury. But working experiences, educational status, and status of the infection prevention committee had not shown any significant association. Those variables were not significant after controlling other variables in multivariate logistic regression analysis. In multivariate logistic regression analysis, the odds of needlestick or sharp injury was 54% less likely in female nurses than male nurses (AOR = 0.461 (95% CI 0.252, 0.845)). Those who worked in the pediatric ward were 68% less likely to get injured by needlestick and sharp objects than those who worked in the surgical ward (AOR = 0.323 (95% CI 0.112, 0.930)). The risk of NSSI was 1.78 times higher in nurses who recapped needles after use than those nurses who had not recapped needles after use (AOR = 1.780 (95% CI 1.025, 3.091)) (Table 4).

## 4. Discussion

In this study, the prevalence of nurses who sustains NSSI in the last 12 months was found to be 36.2% (95% CI 30.2%, 42.3%). This is in line with the studies done in Jimma University Hospital, Ethiopia (39.3%) [50]; India (33.3%) [17]; Tehran (41%) [19]; Southern Ethiopia (42.1%) [51]; Malaysia (31.6) [52]; Iran (39.4%) [53]; and Bahir Dar, Northwest Ethiopia (31.0%) [54]. The current study demonstrated that the magnitude of NSSI was higher than studies done in South Africa (18.8%, 23.5%) [31, 55]; Awi zone, Ethiopia (18.7%) [56]; Nigeria (23.1%) [57]; Tigray, Northern Ethiopia (25.9%) [58]; Bahir Dar (29%) [59]; Malaysia (23.5%, 24.6%, and 9.8%) [60–62]; Kenya (19%) [27]; United Arab Emirates (19%) [63]; East Gojjam, Ethiopia (22.2%) [64]; and Switzerland (9.7%) [65]. It could be due to the difference in the study health facility setups so that the number of screening, diagnostic, follow-up, and other intervention procedures that use needles and medical sharp materials was less in health centers and even the year of the study. But whatever is the difference in the proportion of needlestick and sharp injuries, nurses are at much higher risk to acquire bloodborne pathogens such as HIV and other infectious diseases through needlestick and sharp injuries.

The prevalence in this study is lower than the figure from earlier studies in Southwest Ethiopia (58.8%) [44]; Sri Lanka (43%) [22]; Thailand Regional Hospital (55.5%) [16]; Iran Shiraz University Hospital (54%) [20]; Nigeria (55.8%) [57]; Dessie, Ethiopia (43%) [66]; Saudi Arabia (50.9%) [67]; Egypt (62.3%, 67.9%) [68]; Sarajevo, Bosnia and Herzegovina (61.1%) [69]; Jimma, Ethiopia (44.12) [70]; Hawassa, Ethiopia (46%) [47]; Pakistan (66%) [71]; and Jordan (67.6%) [21]. However, this result is much lower when compared with a study done in Bahir Dar, Ethiopia (66.6%) [37]; South Korea (74.4%) [14]; Pakistan (67%) [15]; and Nepal (74%) [18]. This difference might be related to the fact that the above studies were conducted by mixing all types of health professionals from hospitals, health centers, and clinics, including sociodemographic/economic status, cultural characteristics of the study participants, sampling method, and sample size. The other possible reason might be related to workload and the availability of resources, as well as the work environment, and related to a different time of recall periods.

In this study, the majority of injuries were slight skin penetration (53.1%) while 32.7% were superficial. Regarding causative tools, the most frequent causative tools were needles (86.3%). The present study revealed that 51% of exposed nurses wash with water after exposure while 44.9% with an antiseptic solution. This was lower than the study conducted in Zagazig University, Egypt, which reported that the severity of penetration of needlestick and sharp injuries during the last year among nurses was superficial (74.24%), and the procedures which mostly exposed nurses were injection and withdrawal of blood (56.06% and 43.18%), respectively, and the procedure taken after exposure showed that all exposed nurses used antiseptic after exposure while half of them let blood flow [1]. But this result is higher than the study done by Jimma University for nurses which showed 25.6% for needles and 23.2% for surgical blades [50]. This variation might be due to different times of the recall period. The current study revealed that the syringe needle was a major cause of the injuries (74.2%). It is much higher as compared to the study done in Tehran (Iran) (46.3%) [19] and Southwest Ethiopia (58.8%) [35]. However, it is consistent with a study done in South Africa (78.3%) [31]. This implies that nurses who had been injured by NSSI might be due to inappropriate needle handling practices. It might also be because the majority of the procedures done for the patients require syringe needles that may put nurses at risk of injuries.

Regarding the frequency of injury, 49.5% of the respondents had experienced injuries at least once in a year. This is a little bit higher than the study done in India (33.3%) [17]. But whatever the difference of the proportions of NSSI, nurses might practice needle recapping after use which may put them at risk of injury. For instance, the prevalence of needle recapping after use in this study was 44%, and of these, nearly one-third (30.5%) was recapping using two hands. The practice of recapping is higher in the studies in Nigeria (35.3%) [72]. In this study, injection (37.7%) and blood withdrawal (24.5%) were the major clinical activities that lead to NSSI in this study. A study conducted in Saudi Arabia showed that most of the injuries occur during injections (31.8%) and drawing of venous blood samples (17.2%) [73], and a study conducted in India showed that the commonest clinical activities to cause NSSIS in that study were blood withdrawal (55%), suturing (20.3%), vaccination (11.7%), and recapping needles after use (66.3%), and according to a study done on Malaysian hospital nurses, 27.2% of NSSI causes were recapping of syringes after use [60]. In this study, 64.9% of nurses were vaccinated against hepatitis B. This result is higher than a study done in Jimma University Hospital which showed that 1.76% of the study subjects were vaccinated for hepatitis B vaccine [50], and a study conducted in Egypt showed that only 6.81% were vaccinated for hepatitis B [1]. This variation may be due to the presence of the vaccine in TASH.

Regarding factors found to be associated with the high prevalence of NSSI. From those factors, being female was significantly associated with NSSI; the odds of needlestick or sharp injury was 54% less likely in female nurses than male nurses (AOR = 0.461 (95% CI 0.252, 0.845)). This is consistent with the report from Southwest Ethiopia [35]. The possible explanations might be because men are less likely to use universal precautions, but further studies are warranted to identify exposure differences. Those who worked in the pediatric ward were 68% less likely to get injured by needlestick and sharp objects than those who worked in the surgical ward: AOR = 0.323 (95% CI 0.112, 0.930). This may be because more advanced procedures and manipulation of the syringe and sharp materials were done in the surgical ward. Respondents who practiced needle recapping were 1.78 times more likely to experience an injury than those who did not recap needles after use: AOR = 1.780 (95% CI 1.025, 3.091). This is supported by previous studies [36].

According to this study, there was also a statistically significant difference of NSSI among nurses working in the presence of contaminated needles and sharp materials in the working places (AOR = 2.052 (95% CI 1.110, 3.791)) than their counterparts. Neither age nor marital status was significant, but training and work experience had a significant effect on the magnitude of NSSI among nurses. This is inconsistent with a study conducted in Malaysia [60]. The possible justification might be due to the setup and working environment safety. A safe working environment reduces the probability of sustained needlestick and sharp injuries.

Current working unit/department (AOR = 0.323 (95% CI 0.112, 0.930)), sex (AOR = 0.461 (95% CI 0.252, 0.845)), presence of contaminated needles and sharp materials in the working area (AOR = 2.052 (95% CI 1.110, 3.791)), and needle recapping (AOR = 1.780 (95% CI 1.025, 3.091)) had a significant association with the occurrence of sharp and needlestick injuries in nurses at *p* < 0.05. This result is almost different with the study conducted in East Gojjam Zone Health Institutions health care workers which showed the following results: infection prevention and safety information access and getting training on infection prevention showed a significant association with the occurrence of needlestick and sharp injuries in health care workers [64]. This difference may be related to the difference of the setups they used (standard precaution guidelines) and the mixing of all types of health professionals from health centers. Taking training on infection prevention was not found to be statistically significant in multivariable analysis in this study. Similarly, this finding goes in line with previous findings in a study in Bahir Dar [59], in which training for workers did not necessarily bring about protection from injury exposure. The reason for this may be because the knowledge gained may not necessarily be transferred into the practice of preventive measures and those who participated in the infection prevention training may be other workers who are working as nursing managers and are not involved in clinical practice. Lastly, the sample size might not be sufficient enough to detect the differences. Besides, the result of this study indicates that the infection prevention committee was not a significant determinant of needlestick and sharp injuries. This study was inconsistent with other previous studies [74]. On the opposite side, a study carried out in Debre Berhan, Ethiopia, revealed that the absence of onsite training on IP and safety practice was the main contributor to occupational exposure [75]. According to a study done in Kenya, previous training on IP was protective [27]. The reason for this difference could be the methodological differences and variation in study recall periods, the difference of the setups they used (standard precaution guidelines), and the mixing of all types of health professionals from health centers. The current study found out that educational status was not significantly associated with needlestick and sharp injuries. However, in a study conducted in Egypt, low education levels were the most significant predictors of needlestick and sharp injuries among nurses [1]. Moreover, the result of this study indicates that work experience was not a significant predictor of needlestick and sharp injuries. This study was inconsistent with other previous studies [21, 76]. Generally, needlestick or sharp injury is significantly different among current working unit/department, sex, presence of contaminated needles and sharp materials in the working area, and needle recapping. For the health care provider, complete surveillance of exposure is necessary for the identification of high-risk activities and environments to define new targets for preventive measures and monitoring of the success and failure of the measure taken.

## 5. Strength and Limitation of the Study

Since the study was conducted among randomly selected nurses, it might be generalized to all nurses who had direct contact with patients or equipment used on patients when working in the study hospital. Some staff could not remember if they sustained needlestick and sharp injuries within the past 12 months, some respondents were not sure if their information was kept secret, and nurses were recruited during their lunchtime and were not comfortable to answer freely. Since participants have been asked a one-year exposure experience, there might be recall bias. Since the study was based on self-reported data in estimating the prevalence of NSSI exposure, a common threat to the validity of the self-report can lead to information bias such as social desirability and recall bias. Besides, a cross-sectional study by its nature cannot establish a definitive cause-and-effect relationship to identify the risk factors.

## 6. Conclusion

This study revealed that more than one-third of the study participants had needlestick and/or sharp injury at least once in the previous 12 months. The proportion of needlestick and sharp injuries in the last year was found to be high. In general, this study revealed that no single factor accounted for the occurrence of NSSI. The presence of contaminated needles and/or sharp materials in the working area and needle recapping after use were positively associated with needlestick/sharp injury, while working in the pediatric ward and being female showed a negative association.

## 7. Recommendation

Based on the findings of this study, the following recommendations are forwarded to MOH Ethiopia, Addis Ababa City Administration Health Bureau, TASH managers, and TASH nursing professionals to reduce the occurrence of NSSI and the consequences of NSSI among nurses working in TASH:

- *TASH Hospital Administrates and Nursing Service Directors*. Strengthened regular provision of information on infection prevention and safety to nurses at all levels to create a safe working environment for nurses. Continuous monitoring of workplace safety and an appropriate sharp disposal system
- *TASH Nurse Professionals*. Practice proper use of safety boxes and personal protective equipment when handling needles and sharps. Nurses involved in safe segregation and disposal of all sharp items immediately in marked containers
- *Health Policymakers*. Formulate strategies to improve the working condition for nursing professionals and increase their adherence to universal precautions
- *MOH*. Continuous monitoring of workplace safety should be ensured by MOH
- *Addis Ababa City Administration Health Bureau*. Regular reporting, follow-up, and evaluation of occupational injury exposure among nurses need to be introduced
- *NGO*. Creating awareness for nursing professionals on safety practices of injection

## Figures and Tables

**Table 1:** Distribution of sociodemographic, working environment, and behavioral characteristics of nurses, TASH, Addis Ababa, Ethiopia, June 2018.

Variables	Characteristics	Frequency (*N* = 268)	Percentage (%)
Age	<25	59	22.0
	25-30	115	42.9
	>30	94	35.1
	Total	268	100

Sex	Male	77	28.7
	Female	191	71.3
	Total	268	100

Religion	Orthodox	187	69.8
	Protestant	40	14.9
	Muslim	35	13.1
	Catholic	6	2.2
	Total	268	100

Marital status	Single	158	59.0
	Married	89	33.2
	Divorced	9	3.4
	Widowed	12	4.5
	Total	268	100

Educational status	Diploma	31	11.6
	BSC	202	75.4
	MSC	35	13.1
	Total	268	100

Work experiences	<5	198	73.9
	5-10	41	15.3
	>10	29	10.8
	Total	268	100

Working departments	Emergency & ICU	38	14.2
	Medical	72	26.9
	Surgical	45	16.8
	OPD	73	27.2
	Pediatric	40	14.9
	Total	268	100

Training	Yes	146	54.5
	No	122	45.5
	Total	268	100

IP committee	Yes	197	73.5
	No	71	26.5
	Total	268	100

PEP	Yes	150	56
	No	118	44
	Total	268	100

HBV vaccination	Yes	174	64.9
	No	94	35.1
	Total	268	100

Department report when NSSI occurs	Yes	173	64.6
	No	95	35.4
	Total	268	100

Recapping of a needle after use	Yes	118	44.0
	No	150	56.0
	Total	268	100

Observe any NSSI on nurses	Yes	193	72
	No	75	28
	Total	268	100

Sharp collection box	Yes	180	67.2
	No	88	32.8
	Total	268	100

Injection equipment	Sterilized and reused	21	7.8
	Single-use	233	86.9
	Autodisposable	14	5.2
	Total	268	100

Recommended practice to prevent NSSI	Yes	192	71.6
	No	76	28.4
	Total	268	100

Dirty sharps in working places	Yes	184	68.7
	No	84	31.3
	Total	268	100

Injection environment	Safe	81	30.2
	Unsafe	187	69.8
	Total	268	100

^∗^OPD = outpatient department; ICU = intensive care units; NSSI = needlestick and/or sharp injuries; TASH = Tikur Anbessa Specialized Hospital; IP = infection prevention; HBV = hepatitis B virus; PEP = postexposure practice.

**Table 2:** Frequency distribution of procedures at which exposure happened and actions taken after exposure happened among the exposed nurses during the last year working at TASH, Addis Ababa, Ethiopia, June 2018.

Procedures at which exposure happened among exposed nurses	*N* = 97	Percent (%)^∗^
Injection	37	38.1
Suturing wound	12	12.4
Sample drawing	24	24.7
Operation	16	16.5
Needle recapping	10	10.3
Sharp disposal	6	6.2
Cleaning and sterilization of instruments	12	12.4
Failing of tools	2	2.1
Actions taken after exposure happened		
Let blood flow	11	11.3
Wash with water	50	51.5
Use antiseptic	44	45.4
Lab investigation	2	2.1
Vaccination	2	2.1
Seroprophylaxis	9	9.3

^∗^Each of the percentages does not add up to 100% because respondents could choose several responses which could be more than one reason.

**Table 3:** Bivariate logistic regression model analysis of factors associated with NSSI, TASH, Addis Ababa, Ethiopia, June 2018.

Variables	Category	NSSI		COR (95% CI)	*p* value
		Yes	No		
Age	<25	21	38	0.932 (0.473, 1.834)	0.838
	25-30	41	74	0.934 (0.530, 1.645)	0.813
	>30	35	59	1	

Sex	Male	39	38	1	
	Female	58	133	0.425 (0.247, 0.731)	0.002^∗∗^

Marital status	Single	53	105	0.707 (0.214, 2.3330)	0.569
	Married	35	54	0.907 (0.267, 3.7086)	0.876
	Divorced	4	5	1.120 (0.196, 6.414)	0.899
	Widowed	5	7	1	

Educational status	BSC	73	129	0.5349 (0.260, 1.101)	0.089^∗^
	Diploma	6	25	0.227 (0.075, 0.688)	0.009^∗∗^
	MSC	18	17	1	

Working departments	Emergency	9	16	0.703 (0.257, 1.923)	0.493
	ICU	6	7	1.071 (0.310, 3.6980)	0.913
	Medical	28	44	0.795 (0.374, 1.6930)	0.553
	Surgical	20	5	1	
	OPD	26	47	0.691 (0.324, 1.476)	0.340
	Pediatric	8	32	0.313 (0.118, 0.827)	0.019^∗∗^

Training	Yes	60	86	1.603 (0.965, 2.663)	0.069^∗^
	No	37	85	1	

Working experiences	<5	71	127	0.599 (0.273, 1.312)	0.200^∗^
	5-10	12	29	0.4439 (0.164, 1.195)	0.108^∗^
	>10	14	15	1	

Dirty sharps in working places	Yes	75	109	1.939 (1.098, 3.423)	0.022^∗∗^
	No	22	62	1	

IP committee	Yes	76	21	1.495 (0.833, 2.684)	0.1778^∗^
	No	121	50	1	

Safety box at right places	Yes	70	121	1.071 (0.616, 1.862)	0.808
	No	27	50	1	

Universal precaution	Yes	72	120	1.224 (0.699, 2.144)	0.480
	No	25	51	1	

Sharp collection box	Yes	66	114	1.065 (0.625, 1.812)	0.818
	No	31	57	1	

Needle recapping	Yes	53	65	1.964 (1.185, 3.255)	0.009^∗∗^
	No	44	106	1	

Injection environments	Safe	32	49	1	
	Unsafe	65	122	1.226 (0.716, 2.098)	0.458

^∗^Significant at *p* ≤ 0.2. ^∗∗^Significant at *p* ≤ 0.05. COR = crude odds ratio; OPD = outpatient department; ICU = intensive care units; NSSI = needlestick and/or sharp injuries; TASH = Tikur Anbessa Specialized Hospital; IP = infection prevention; HBV = hepatitis B virus; PEP = postexposure practice.

**Table 4:** Logistic regression model analysis of factors associated with NSSI, TASH, Addis Ababa, Ethiopia, June 2018 (*N* = 268).

Variables	Category	NSSI		AOR (95% CI)	*p* value
		Yes	No		
Sex	Male	39	38	1	
	Female	58	133	0.461 (0.252, 0.845)	0.012^∗^

Working departments	Emergency	9	16	0.670 (0.226, 1.984)	0.469
	ICU	6	7	0.8464 (0.214, 3.487)	0.838
	Medical	28	44	0.666 (0.288, 1.542)	0.343
	OPD	26	47	0.711 (0.299, 1.692)	0.441
	Pediatric	8	32	0.323 (0.112, 0.930)	0.036^∗^
	Surgical	20	25	1	

Educational status	BSC	73	129	0.460 (0.172, 1.234)	0.123
	Diploma	6	25	0.256 (0.063, 1.048)	0.058
	MSC	18	17	1	

Dirty sharps in working places	Yes	75	109	2.052 (1.110, 3.791)	0.022^∗^
	No	22	62	1	

Work experiences	<5	71	127	1.413 (0.520, 3.839)	0.498
	5-10	12	29	0.602 (0.202, 1.795)	0.363
	>10	14	15	1	

IP committee	Yes	76	121	1.702 (0.878, 3.299)	0.116
	No	21	50	1	

Needle recapping	Yes	53	65	1.780 (1.025, 3.091)	0.041^∗^
	No	44	106	1	

Training	Yes	60	86	0.753 (0.429, 1.323)	0.324
	No	37	85	1	

^∗^Significant at *p* ≤ 0.05. AOR = adjusted odds ratio; OPD = outpatient department; ICU = intensive care units; NSSI = needlestick and/or sharp injuries; TASH = Tikur Anbessa Specialized Hospital.

## Data Availability

All data about this study are contained and presented in this document.

## Abbreviations

AAU: Addis Ababa University
AIDS: Acquired immunodeficiency syndrome
AOR: Adjusted odds ratio
BSC: Bachelor of Science
CHS: College of Health Sciences
CI: Confidence interval
COR: Crude odds ratio
GC: Gregorian calendar
HBV: Hepatitis B virus
HCV: Hepatitis C virus
ICN: International Council of Nurses
HIV: Human immunodeficiency virus
NSI: Needlestick injury
NSSI: Needlestick and sharp injury
TASH: Tikur Anbessa Specialized Hospital
USA: United States of America
WHO: World Health Organization.
